# Geographical Clustering of High Risk Sexual Behaviors in “Hot-Spots” for HIV and Sexually Transmitted Infections in Kwazulu-Natal, South Africa

**DOI:** 10.1007/s10461-013-0578-x

**Published:** 2013-08-10

**Authors:** Gita Ramjee, Handan Wand

**Affiliations:** 1 HIV Prevention Research Unit, Medical Research Council, 123 Jan Hofmeyr Road, Westville, Durban, South Africa; 2 The Kirby Institute, University of New South Wales, Sydney, NSW Australia; 3 Department of Epidemiology and Population Health, London School of Hygiene and Tropical Medicine, London, UK

**Keywords:** Sexual transmitted infections and risk behaviours, Spatial clustering, South Africa

## Abstract

We investigated geographical variations of three sexually transmitted infections (STIs) namely chlamydia, gonorrhea and syphilis in the greater Durban area, so as to optimize intervention strategies. The study population was a cohort of sexually active women who consented to be screened in one of three biomedical studies conducted in Durban. A total of nine local regions collectively formed three clusters at screening, five of which were previously defined as HIV hot-spots. STI cases were geo-coded at the census level based on residence at the time of screening. Spatial SaTScan Statistics software was employed to identify the areas with a disproportionate prevalence and incidence of STI infection when compared to the neighboring areas under study. Both prevalence and incidence of STIs were collectively clustered in several localized areas, and the majority of these locations overlapped with high HIV clusters and shared the same characteristics: younger age, not married/cohabitating and multiple sex partners.

## Resumen

Se investigaron las variaciones geográficas de los tres infecciones de transmisión sexual (ITS), es decir, la clamidia, la gonorrea y la sífilis en el área metropolitana de Durban, con el fin de optimizar las estrategias de intervención. La población de estudio fue una cohorte de mujeres sexualmente activas que dieron su consentimiento para someterse a las pruebas en uno de los tres estudios biomédicos realizados en Durban. Un total de nueve regiones locales forman colectivamente tres grupos de investigación, cinco de los cuales fueron definidos previamente como VIH puntos calientes. Casos de ITS fueron geo-codificados a nivel censal basada en la residencia en el momento de la detección. Software Espacial Scan Estadísticas (SaTScan) fue empleado para identificar las zonas con una prevalencia desproporcionada e incidencia de ITS en comparación con las zonas vecinas en estudio. y la incidencia. ITS se agrupan colectivamente en varias zonas localizadas, y la mayoría de estos lugares se superponen con los clústeres de alta de VIH y comparte las mismas características: la edad más joven, no está casado/cohabitan y múltiples parejas sexuales.

## Introduction

South Africa has the highest burden of human immunodeficiency virus (HIV) infection in the world with an estimated 5.6 million people infected in 2011 [1]. Other sexually transmitted infections (STIs), particularly chlamydia, gonorrhea and syphilis are also reported to cause morbidity in the region and have been linked to HIV transmission [2–5]. Unprotected heterosexual exposure has been reported to be the primary mode of HIV and STI transmission in this region with young sexually active adults being the most vulnerable population prone to these infections [6]. Additionally, recent epidemiological studies indicate a high incidence of both HIV and STIs among women, with low condom use being a contributing factor to transmission of infections [7, 8].

We have previously highlighted the importance of geographic clustering in the potential transmission of HIV infection [9, 10]. The current study represents a continuation of our previous work focusing on the identification of “high STI” in specific geographical areas.

We hypothesized that geographical clusters of STIs would potentially represent sexual networks that could overlap with the areas identified as hot-spots for HIV infections. We also investigated if demographic or sexual behavioural data could further differentiate the geographically distinct STI clusters in this region. If detected, these clusters may potentially represent relatively homogenous individuals, by splitting a large sexual network into smaller communities.

## Methods

### Study Areas and Geographical Data

Data from 5,753 sexually active women who consented to screening for three studies from six clinics and 158 census locations were combined for this study. The studies were as follows: the methods for improving reproductive health in Africa (MIRA) trial of the vaginal diaphragm for HIV prevention, took place at two sites between September 2002 and September 2005 (rural Umkomaas, 44 km south of Durban, and Botha’s Hill, 31 km west of Durban) [11]; the Microbicides Development Programme (MDP) feasibility study in preparation for Phase III microbicide trials, took place at two sites between August 2002 and September 2004 (semi-rural Tongaat, 31 km north of Durban, and Verulam, 22 km north of Durban) [12]; and the HIV Prevention Trials Network (HPTN 055) site-preparedness study for future implementation of Phase II/IIb/III clinical trials, took place at two sites between May 2003 and January 2005 (rural district of Hlabisa and urban Durban)[13].

### Study Procedures

For the MIRA and HPTN 055 studies, HIV diagnostic testing was achieved using two rapid tests on whole blood sourced from either finger-prick or venepuncture (Determine HIV-1/2, Abbot Laboratories, Tokyo, Japan and Oraquick, Orasure Technologies, Bethlehem, PA, USA). For the MDP feasibility study, the Abbot IMX ELISA test (Abbot Diagnostics, Africa Division), in combination with the Vironostika HIV1/2 ELISA for positive and equivocal results, was used on whole blood sourced from venepuncture. In the MIRA trial, a first catch urine sample was collected for DNA PCR testing for chlamydia and gonorrhoea (Roche Pharmaceuticals, Branchberg, NJ, USA) at screening, while a blood sample was collected for syphilis [rapid plasma regain (RPR) and confirmatory TPHA (Omega Diagnostics, Alva, UK)]. In the HPTN 055 study, urine samples were collected for diagnosis of gonorrhoea and chlamydia using the BD Probe Tec ET assay (Becton–Dickinson, Sparks, MD). Syphilis was tested using RPR (BD Macro-Vue) and confirmed using TPHA (Serodia Fujirebio). The MDP feasibility study tested for chlamydia and gonorrhoea using PCR (COBAS Amplicor, Roche Molecular Diagnostics, Pleasanton, CA, USA); syphilis was tested by RPR and confirmatory TPHA (Omega Diagnostics, Alva, UK). Only women who had test results and geographical data were included in the study. At all visits, all participants received a comprehensive HIV prevention package consisting of HIV pre- and post-test counseling, intensive STI/HIV risk reduction counseling and treatment of curable, laboratory-diagnosed STIs. The studies were all approved by the local biomedical research ethics committee of the University of KwaZulu-Natal, Durban.

### Detecting and Characterizing Clusters

The spatial scan statistics (SaTScan) [14–16] software (version 9.1, www.satscan.org) was used to detect the potential excess of STIs [17, 18]. Briefly, scan statistics were calculated at each geographical location to assess the number of excess STIs in the target population. Geographic data such as latitude and longitude for location were used as a proxy for residential area of women.

Stata (Version 12.0, CS, TX) software was also used to compare the characteristics of each cluster area with a non-cluster area. Chi square and Fisher’s exact tests were used to compare differences in proportions.

## Results

### Characteristics of Study Subjects

As described previously [9], the current study included data from the six clinical sites and 158 census locations where the predominantly black population was estimated to be approximately 2,400,000 (http://www.statssa.gov.za/publications/populationstats.asp). The geographical data of a total of 728 women who tested positive for any one of the STIs namely, chlamydia, gonorrhea and syphilis, at screening or enrolment were used to identify areas of high STI prevalence. Additionally, 680 women who were STI negative at screening but who tested positive during study follow-up were included in the analysis.

### Hot-Spots of Increased STI Prevalence

The SaTScan analysis for significant spatial clustering for excess STI prevalence, after adjusting for the size of the study population and women’s age, is represented in Table 1. The analysis identified three clusters of high prevalence when compared to other study sites. These clusters included 175 cases (24 % of all cases) recruited from two study sites: a less urbanized clinic in Botha’s Hill and a peri-urban clinic in Umkomaas. The first cluster, which was within a 2.47 km radius in the south of Durban, had 78 cases from four residential areas: Umkomaas, Naidooville, Mkomazi and Saiccor (relative risk, RR = 10, *p* = 0.001). The second cluster involved 86 cases (RR = 8, *p* = 0.001) centered within 9.76 km radius and located exclusively west of Durban from four residential areas: Inchanga, Hammarsdale, Botha’s Hill and Cato-Ridge. The final cluster was made up by one residential area north-west of Durban, Molweni, which had 11 STI cases (RR = 51, *p* = 0.001).

**Table 1:** Distribution of STI infection cases by districts at screening (age and population adjusted)

Clusters	Radius (km)	Observed STI cases	Relative risk of excess STI cases	Test statistics^a^	*p* value	Total locations
Cluster 1	2.47	78	10	212.11	0.001	3
Cluster 2	9.76	86	8	29.38	0.001	4
Cluster 3	0	11	51	19.23	0.001	1

1. Umkomaas, Naidooville, Mkomazi Drift and Saiccor; 2. Inchanga, Hammersdale, Botha’s Hill, Cato-Ridge; 3. Molweni

^a^From likelihood-ratio test

### Hot-Spots of STI Incidence

A total of 2,523 HIV negative and STI negative (or treated) women enrolled in the three studies with a median duration of 12 months follow-up. Of these, 680 had tested positive at least once during the follow-up period (incidence rate of 20 per 100 person years). Using the SaTScan programme, and adjusting for the size of the population and women’s age, a total of 126 (19 % of the all STIs) of the women who tested positive for at least one STI during the follow-up period were geographically clustered into five hot-spots (Table 2). The highest incidence of STIs was observed in a cluster that comprised two census areas south of Durban, namely Umkomaas and Mkomazi, encompassing a radius of 2.64 km (RR = 12, *p* < 0.001). The second cluster was centred within ~6.5 km radius and included Inchanga, Hammarsdale (RR = 24, *p* < 0.001); Cato-Ridge, Molweni and Botha’s Hill were all identified to be the third, fourth and fifth clusters respectively.

**Table 2:** Distribution of STI incidence by districts during the follow-up

Clusters	Radius (km)	Observed STI incidence cases	Relative risk of excess HIV cases	Test statistics^a^	*p* value	Total locations
Cluster 1	2.64	68	12	51.86	0.001	2
Cluster 2	6.48	32	24	37.54	0.001	2
Cluster 3	0	11	18	27.16	0.001	1
Cluster 4	0	7	51	17.41	0.001	1
Cluster 5	0	8	9	8.03	0.002	1

1. Umkomaas, Mkomazi; 2. Inchanga, Hammersdale; 3. Cato-Ridge; 4. Molweni; 5. Botha’s Hill

^a^From likelihood-ratio test

### STI Hot-Spots in Relation to HIV Hot-Spots

Both previously reported HIV and the current STI studies consistently determined to be geographical locations in “west of Durban” and “south of Durban” with excess HIV and STI cases which broadly overlapped. Demographic characteristics and sexual risk behaviour of women who were living in the cluster areas (i.e. hot-spots) were compared with those who did not (Table 3).

**Table 3:** Selected characteristics of the hot-spots

Characteristics	Percentage (%)	HIV/STI hot-spots (%)	Rest of the study sites (%)	Test statistics^a^	*p* value
Age groups (years)				41.41	<0.001
<25	41	59	40		
25–34	37	31	37		
34+	22	10	23		
Language spoken				68.41	<0.001
English	12	19	9		
Zulu and other	88	81	91		
Religious				0.0192	0.890
Christian	91	91	91		
Other	9	9	9		
Age at sexual debut				2.02	0.155
<16	21	20	22		
16+	79	80	78		
Marital status				10.95	0.001
Not married	85	92	84		
Married	15	8	16		
Living with a regular partner				22.52	<0.001
No	70	82	69		
Yes	30	18	31		
Lifetime number of sexual partners				9.74	0.002
Less than three	53	43	53		
Three or more	47	57	47		
Current contraceptive use				2.08	0.198
Injectables/pills	42	42	42		
Male/female condoms	26	27	25		
Others (e.g. none, traditional)	32	30	33		

^a^From Chi square test

Compared to those who were living outside the HIV/STI geographical hot-spots, women who were living in HIV/STI hot-spots were significantly younger (age <25, 59 vs. 40 %, *p* < 0.001), not married (92 vs. 84 %, *p* = 0.001), not cohabiting with their regular sex partners (82 vs. 69 %, *p* < 0.001) and they also reported a higher number of lifetime sexual partners (3+, 57 vs. 47 %, *p* = 0.002). A higher number of women in the HIV/STI hot-spots reported English as their preferred language (19 vs. 9 %, *p* < 0.001). Women were similar in terms of religion, age at sexual debut and type of contraceptive use regardless of whether they were living in one of the hot-spots or not.

**Fig. 1:**
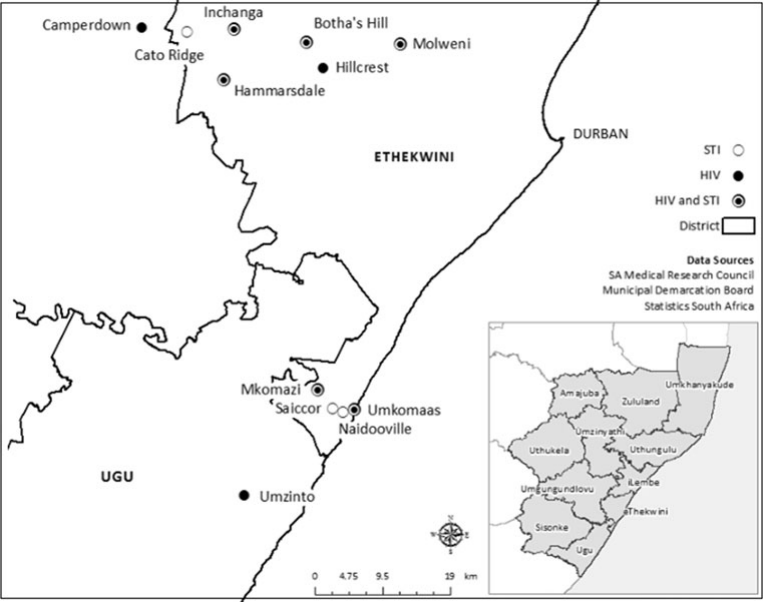
HIV/STI hot-spots

Figure 1 shows the location of the excess STI and HIV cases. A total of nine local regions collectively formed three clusters, of which five of them were also previously defined as HIV hot-spots [9].

## Discussion

Building on our previous data of identifying geographic excesses of HIV infections at rates among the highest in the world in certain rural communities of Durban, we have now identified excess cases for STIs with overlapping areas of excessive HIV infection. Additionally, the current study has identified three statistically significant areas of excess STI prevalence and incidence rates around the Umkomaas clinic (44 km south of Durban) and Botha’s Hill clinic (31 km west of Durban) with the inference that risks for chlamydia, gonorrhea and syphilis are associated with statistically definable socio-geographic locations. In terms of spatial persistence, these cluster areas were overlapped with the high HIV clusters (prevalence and incidence).

Determining the geographical variations of HIV and STIs is critical for targeting areas most in need of prevention interventions. Our study confirmed that “high transmission areas” overlap with high prevalence and incidence of HIV and STI cases. These high transmission areas are located predominantly in areas where HIV prevalence is higher than in neighbouring areas.

Our finding suggesting that the clusters for STIs overlap with HIV clusters is hardly surprising. Others have reported overlap of HIV/STI prevalence previously [19]. Finding overlapping areas of HIV and STI’s is not unusual as both these sexually transmitted infections share common demographic, social and sexual behavioural risk factors [20]. Furthermore it has been well documented that curable STIs are risk factors for HIV [21, 22]. For this reason, STI diagnosis and treatment is always included as part of HIV prevention package.

Our study has several limitations that need to be acknowledged. Due to the nature of the research conducted in these clinical trials, the study population selected could potentially be at higher risk for infection with HIV and other STIs. Therefore, the study population may not be representative of all women in the KwaZulu-Natal province. Furthermore, only six study sites were included in this analysis, which may not be fully representative of the region itself. We also cannot rule out the possibility that the findings could be due in part to unmeasured characteristics, such as commercial sex work or population migration.

## Conclusion

Our studies of spatial and demographic variation in prevalence and incidence of STI infection suggested areas of high sexual networking, with poor condom use among young women in these communities. Data from our previous studies suggested that young women are particularly at high risk of both HIV and STI acquisition. Although South Africa has a generalised epidemic, there are pockets in communities which demonstrate concentrated epidemics. Identification of these pockets of “hot-spots” is critical for targeted biomedical behavioural and structural intervention to reduce the burden of HIV/STI in the communities.
